# The species range‐size patterns for vascular plants of Seorak Mountain (Korea): Relationship between group of life forms and phytogeography affinity along the elevational gradient

**DOI:** 10.1002/ece3.8033

**Published:** 2021-08-24

**Authors:** Mi‐Hyun Lee, Ju Hyeon Song, Seong Yeob Byeon, Jeong Eun Lee, Ho Jin Kim, Seung‐Beom Chae, Chung Weon Yun, Ji‐Dong Kim

**Affiliations:** ^1^ Baekdudaegan National Arboretum Bonghwa Korea; ^2^ Korea Institute of Arboretum Management Sejong Korea; ^3^ Department of Forest Science Kongju National University Yesan Korea

**Keywords:** elevation gradient, elevational Rapoport rule, phytogeography, Seorak Mountain, species diversity

## Abstract

Research on species richness patterns and the advanced elevational Rapoport rule (ERR) has been widespread in recent years; however, there is a lack of such research for the temperate mountainous regions in northeast Asia. Here, we collected plant species from the Seorak Mountain in northeast Asia through field surveys. The species were divided into 11 groups according to the life‐form types and phytogeography affinities of each species. The ERR was evaluated using Steven's method and by examining the species richness patterns of each group. The species richness patterns revealed a positive multimodal pattern along the elevation gradient, but phytogeography affinities (increasing trend) and life‐form analysis (unimodal) exhibited different patterns. The elevation gradients (1,350 m for the mean elevation–range relationships), which are affected by the boundary effect and different life forms, did not consistently support the ERR. However, herbs as well as rare, endemic, and red list species showed consistent support for the ERR, which could be attributed to the influence by phytogeography affinities. Therefore, the results from Seorak Mountain showed that the ERR was not consistent for different plant life forms in the same area; however, phytogeography affinities could support and explain ERR.

## 1 INTRODUCTION

The birth and history of life in nature are not random. The concept of species, the lowest unit in biological classification, contains various information. Among them, the range size of species varies among taxa, such as mammals, insects, birds, and plants (Brown et al., 1996; Gaston, 1998). They show distinct patterns based on the evolutionary history and ecological requirements of the taxa, their habitat, and geographic conditions (Hernández‐Rojas et al., 2020; Kreft et al., 2010; Lomolino et al., 2006; Smith, 1993). These patterns are reported for a wide range of taxa in many regions (Addo‐Bediako et al., 2000; Kim, Seo, et al., 2019; Morin & Lechowicz, 2011; Pintor et al., 2015; Ribas & Schoereder, 2006; Tomašových et al., 2016); however, the interaction between the taxon range size and species diversity patterns from various regions lacks empirical evidence.

Biodiversity, which is continuously being explored worldwide, remains obscure. In addition, the biodiversity of many regions remains unexplored. Evaluating the patterns of species richness according to the altitude gradients in the unexplored regions is very important for the conservation of biodiversity. Biodiversity patterns along elevation gradients have been studied for numerous taxa and terrain extents (Feng et al., 2016; Rahbek, 1995, 1997; Stevens, 1992; Vetaas & Grytnes, 2002; Wang et al., 2007; Wu et al., 2014; Zhou et al., 2019). In general, positively unimodal and/or monotonically declining are the most common patterns of vertical richness along elevation gradients of mountains (Pan et al., 2016; Rahbek, 1995, 2005; Wang et al., 2007). The former pattern indicates that the species richness increases first, decreases after the mid‐peak, and maximum diversity occurs below the middle of the elevation gradients (Kessler, 2000; Trigas et al., 2013; Vetaas & Grytnes, 2002). Additionally, few other patterns of increase in species richness along the elevation gradients, such as increasing, horizontal, and decreasing patterns, have been reported.

Among the various theories on biodiversity patterns, Stevens (1992) elevational Rapoport rule (ERR) remains widely debated (Almeida‐Neto et al., 2006; Colwell & Hurtt, 1994; Fleishman et al., 1998; Ogwu et al., 2019). This approach has attracted the attention of ecologists and biogeographers worldwide; however, there is a considerable controversy regarding the ERR, because of the high level of variability supporting the hypothesis. Some studies partially support the ERR (Chan et al., 2016; Feng et al., 2016; Rohner et al., 2015; Sanders, 2002; Zhou et al., 2019), while the others offer little or no support (Bhattarai & Vetaas, 2006; Fleishman et al., 1998; Guerrero et al., 2011; Külköylüoğlu et al., 2012; Kwon et al., 2014; McCain & Bracy Knight, 2013). Altitude range shifts due to climate change can increase the risk of extinction for range‐restricted species (Elsen & Tingley, 2015; Freeman & Class Freeman, 2014; Harte & Shaw, 1995; McCain & Colwell, 2011). Therefore, the accumulation of research and information on ERR in various regions helps to increase our understanding of the rule; it also aids the conservation of species with particularly narrow distributions, in turn helping maintain and promote biodiversity.

An important prediction of the ERR is the positive relationship between range size and elevation (Stevens, 1992). The pattern in the altitude range size may differ for each taxon (Feng et al., 2016; Gaston, 1996), suggesting that the range altitude relationship varies depending on the ecological characteristics and physiological adaptations to the climate or microenvironment along the altitude gradient. Temperate taxa show a wider altitude range size, compared to that of tropical taxa, because they may have experienced higher variability in environmental factors during their evolutionary and geographic history (Oommen & Shanker, 2005; Wang et al., 2007). Therefore, information from various regions is required to determine the link between ecological and physiological properties and biogeographic affinity.

Another hypothesis made using the ERR is that species diversity decreases as altitude increases. However, the results from previous studies have been inconsistent (Feng et al., 2016; Zhou et al., 2019). For ERR, a multimodal trend, rather than the unimodal decreases, has been observed worldwide; and therefore, further surveys are required. Species with tropical affinities can migrate to other habitats along the warm climate zones (Bergamin et al., 2021; Feeley et al., 2012). This suggests that biogeographic affinity of different taxa is capable of differentiated adaptation to environmental factors, including the altitude. Species with different biogeographic affinities may exhibit varying patterns of richness with altitude, which explains the differences in support for the richness‐altitude hypothesis of the ERR (Feng et al., 2016; Zhou et al., 2019).

It is unclear whether ERR accounts for the relationship between the various life forms and phytogeographic affinities (Zhou et al., 2019). Phytogeographic affinities are linked to elevation range sizes and elevation trends (Wang et al., 2007). The hard boundary effect is bounded by the upper and lower limits (boundaries) of the altitude according to the species range size, and the unimodal pattern of species diversity occurs as an overlapping increase of the species range size along the mid‐peak of elevation gradients (Feng et al., 2016). Therefore, exploration of new regions is essential for comparing differences in ERR with respect to the influence of life forms and phytogeographic affinities.

In this study, (a) the ERR was applied to Seorak Mountain (1,708 m), which is relatively lower than the world's highest mountains (Himalayas at 8,848 m or Andes at 6,961 m); (b) we determined the plant distribution pattern according to the boundary effect and plant geographical affinity; (c) information regarding the species range‐size distribution patterns on the Seorak Mountain area was obtained; and (d) based on our findings, we hope to highlight the importance of exploring the biodiversity patterns in this area, which is unexplored.

## 2 MATERIALS AND METHODS

### 2.1 Study area

**FIGURE 1:**
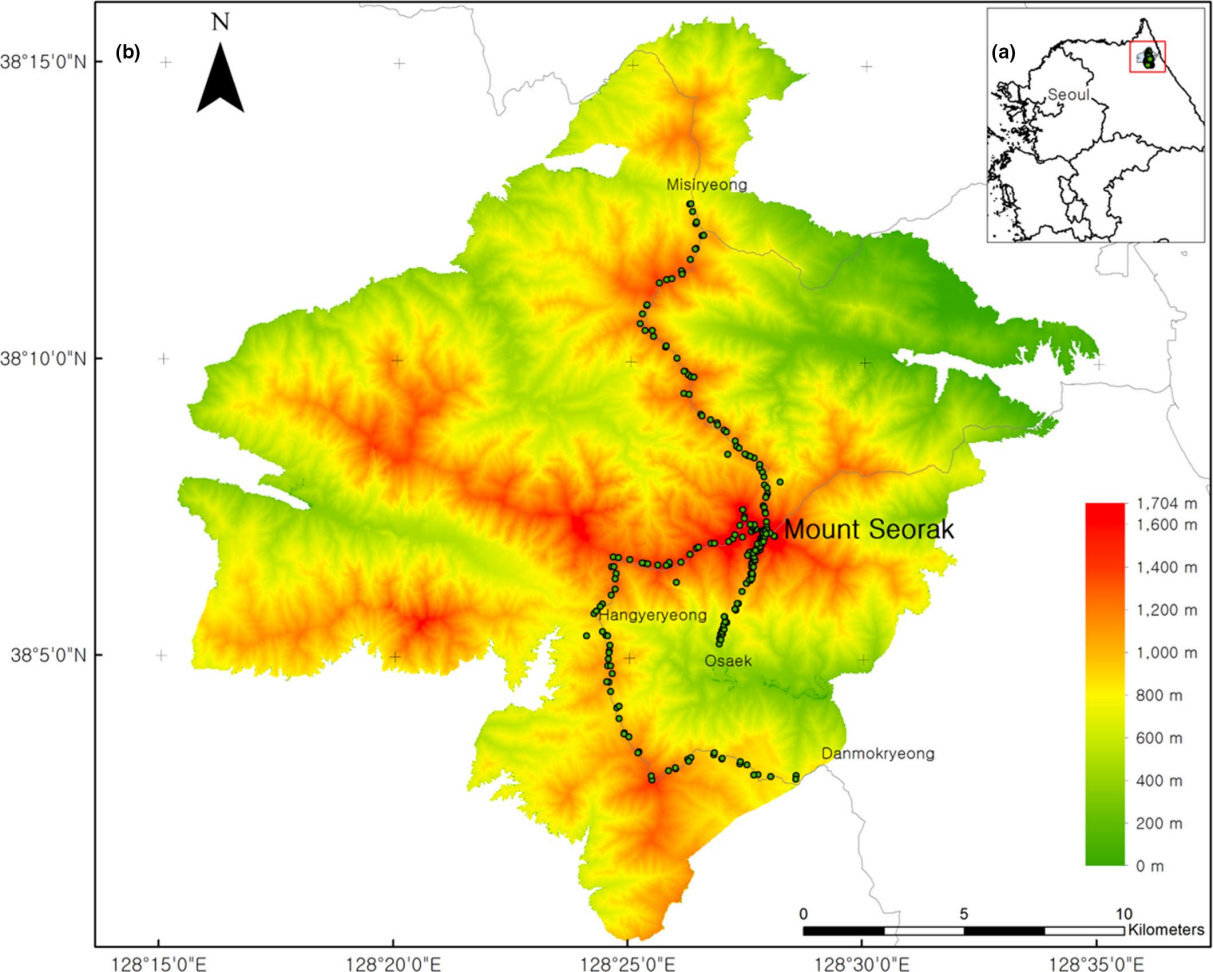
(a) Location of the Seorak Mountain (peak 1,708 m) in Korea; (b) 100 m vertical elevation bands of the Seorak Mountain. The survey route and plots were as follows (1) Misiryeong, (2) Hangyeryeong, (3) Danmokryeong, and (4) Osaek

The Seorak Mountain (128°18′N, 38°05′E) is in eastern Korea and covers an area of approximately 398 km^2^ (Figure 1a). The main peak of Seorak Mountain is Daecheongbong (1,708 m), and it is the second highest peak in Korea. The climate of the region is temperate, with a mean annual temperature of 3.05°C and mean annual precipitation of 1,537.39 mm (Kim, Lim, et al., 2019). Its temperate forests comprise of *Pinus densiflora* or *Abies holophylla* in the lowlands and *Betula ermanii*, *Pinus koraiensis*, *Quercus mongolica*, and *Abies nephrolepis* in the highlands. There are also dwarf tree species near the peak and in the highlands, including *Pinus pumila*, *Taxus caespitosa*, and *Thuja koraiensis*, as well as arctic‐alpine plants, such as *Arctous ruber*, *Crataegus komarovii*, and *Vaccinium uliginosum*.

### 2.2 Data collection

To evaluate the relationship between elevation range size and species richness of vascular plants along the elevation gradients, the elevation range (500–1,708 m) of investigation was chosen. This study area was divided into 13 elevation bands for establishing the field survey route (100 m bands, Figure 1b). The survey area was chosen considering the various terrains and environmental features (e.g., topography, valleys, ridges, and slope direction), within each elevation band. We conducted surveys in multiple elevation bands to minimize the possibility of bias due to uneven sampling. In addition, field surveys were conducted using the same sampling intensity via a phytosociology‐based plot sampling method. Each plot (400 m^2^) was installed on the mountain at elevation intervals of 100 m using the Misiryeong, Hangyeryeong, Danmokryeong, and Osaek trails on Daecheongbong. The total number of survey plots was 228. The lengths of the Misiryeong, Hangyeryeong, Danmokryeong, and Osaek trails are approximately 15.4, 9.5, 15.5, and 10.3 km, respectively. The study was carried out from March to October in 2016 to 2020. Within each plot, the cover‐abundance scale and plant species were recorded using the vegetation survey method (Braun‐Blanquet, 2013). The location of each plot was recorded using Garmin Montana 64s GPS equipment (Garmin).

### 2.3 Plant life forms and taxonomy

Following the Raunkiær system (Raunkiær, 1934), each species was classified as tree, shrub, liana, herb, pteridophyte, or woody species (including trees, shrubs, and lianas) based on the species descriptions in the illustrated plant books by Lee (2003) and KNA (2008, 2010). Species were classified as common, rare, or endemic to Korea (KFS, 2010a, 2010b; KNA, 2008, 2010; Lee, 2003). In addition, rare plants were classified into different groups based on the IUCN red list 2020. The rare and red list species found in Mountain Seorak have phytogeographic affinities (e.g., *P. pumila*, *Leontopodium leiolepis*, *A. ruber*, and *Thalictrum coreanum*). In Korea's Seorak Mountain, native species or rare species appear in isolation after the Pleistocene Epoch (Chung et al., 2017; Kim, Lim, et al., 2019; Kong, 2002, 2004). Rare or endemic plant species appearing in Seorak Mountain are considered tentatively as having a phytogeographical affinity, and most of the polar‐alpine plants appearing in Korea are isolated and distributed at elevations of 1,500–1,800 m or higher. In particular, Seorak Mountain is a mountainous area that exhibits these phytogeographical affinities; it has formed a treeline from 1,500 m and has an extreme climate and environment. With this phytogeographic perspective, a species list was prepared for the endemic or rare plants, and red list species recorded in the field survey.

### 2.4 Species richness

Species richness was defined as the total number of species in all the selected plots within the 100‐m elevation band, referred to as gamma diversity; a species was defined as being present in every 100 m band between its upper and lower elevational limits (Bhattarai & Vetaas, 2006; Feng et al., 2016; Stevens, 1992; Vetaas & Grytnes, 2002; Zhou et al., 2019). We calculated the species richness for the distribution patterns of the total plant species, each life form, and each rare, endemic, and red list species (IUCN, 2020). To explain the potential basic mechanism that induced the diversity pattern of the species in Seorak Mountain, we analyzed the elevation pattern at the family level along the elevation gradients.

### 2.5 Elevation range size

The number of species present in each plot was estimated using the interpolation method. To estimate the range‐size distribution of each species, we identified the minimum and maximum elevation for the distribution of each plant species in each 100 m elevation band. Species that occurred only in a single plot were given a range of 100 m and included in the analysis, referred to as gamma diversity. We calculated the elevation patterns of the species richness, each life form, and each group of phytogeographic affinities (i.e., endemic or rare plants, and IUCN red list). The mean elevational range of the species in a given plot was calculated by averaging the elevational ranges of each species present (Stevens, 1992).

We used our own field observations based on Steven's method and generalized additive models to explore the elevational pattern of range size, calculated using the *gam* function of the R package *ggGam* (Feng et al., 2016; Zhou et al., 2019). The elevation range size for each species was estimated using the distribution patterns between the minimum and maximum elevations. A cubic smooth spline was used to evaluate the significance of specific trends in the elevation range size; and species richness was calculated using the *plot_smooths* function of the R package *mgcv* (Feng et al., 2016; Hastie & Tibshirani, 1990; Zhou et al., 2019). These analyses were carried out using R 3.6.3 (R Core Team, 2020).

## 3 RESULTS

### 3.1 Patterns of species richness along the elevation gradient

**FIGURE 2:**
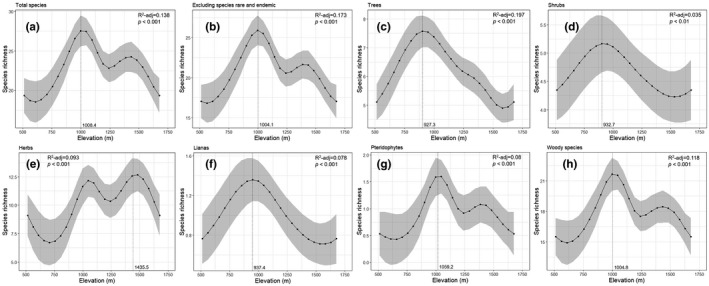
Elevation patterns of species richness of vascular plants on the Seorak Mountain. Total species richness (a); species richness excluding rare, endemic, and red list species (b); and species richness of trees (c); shrubs (d); herbs (e); lianas (f); pteridophytes (g); and woody species (h)

A list of 238 plant species including varieties and subspecies belonging to 163 genera and 70 families was compiled for Seorak Mountain, based on field data collected during vegetation surveys in this region from 2016 to 2020 during the most favorable season for plant flowering (i.e., when most plants could be identified). The total species richness exhibited a positively multimodal pattern along the elevation gradient, with a pronounced mid‐peak at 1,008.4 m above sea level (a.s.l.). At this peak, 41 taxa were identified in each elevation (Figure 2a). After rare and endemic species were excluded, the results were similar to those of the pronounced mid‐peak at 1,004.1 m a.s.l; 39 taxa were observed at the peak (Figure 2b). For the different life‐form groups, there were decreases in tree species at 927.3 m, shrub species at 932.7 m, and climber species at 937.4 m (Figure 2c,d,f). In contrast, the multimodal pattern exhibited decreased herb species at 1,435.5 m, pteridophytes at 1,104.1 m, and woody species (including trees, shrubs, and lianas) at 1,004.8 m (Figure 2e,g,h).

**FIGURE 3:**
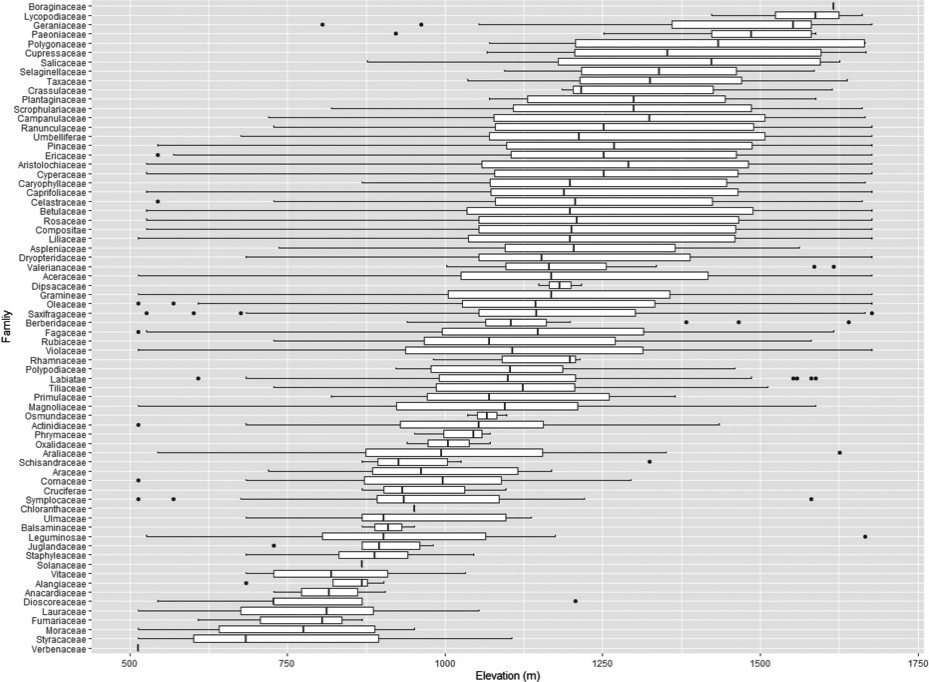
The richness of families within each plot in the vegetation established at the study sites, Seorak Mountain

The analysis at the family level along the elevation pattern indicated that the number of families increased with the elevation. In addition, the average distribution of families containing species with a wide altitude range such as Viloaceae, Aceraceae, Liliaceae, Rosaceae, and Betulaceae was found to be between 900 and 1,100 m (Figure 3).

### 3.2 Mean elevation range size for the distribution of life‐form groups

**FIGURE 4:**
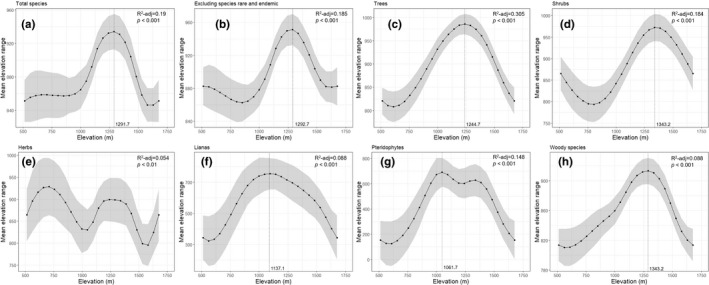
Mean elevation range size of different of life‐form groups along the elevation gradient of the Seorak Mountain: (a) total plants; (b) total plants excluding rare, endemic, and red list species; (c) trees; (d) shrubs; (e) herbs; (f) lianas; (g) pteridophytes; and (h) woody species

The mean elevation range size for total species richness exhibited a pronounced downward trend after the mid‐peak at 1,291.7 m (Figure 4a). The mean elevation range size of excluding rare, endemic, and red list species showed a downward trend at 1,292.7 m in the elevation regions (Figure 4b), as observed for total species richness. Similarly, trees, shrubs, lianas, and woody groups exhibited a sharp downward trend after the mid‐peak. Pteridophytes exhibited a gentle downward trend at 1,061.7 m, but no trend was detected for herbs (Figure 4).

### 3.3 Elevation patterns of the rare, endemic, and red list species (IUCN)

**FIGURE 5:**
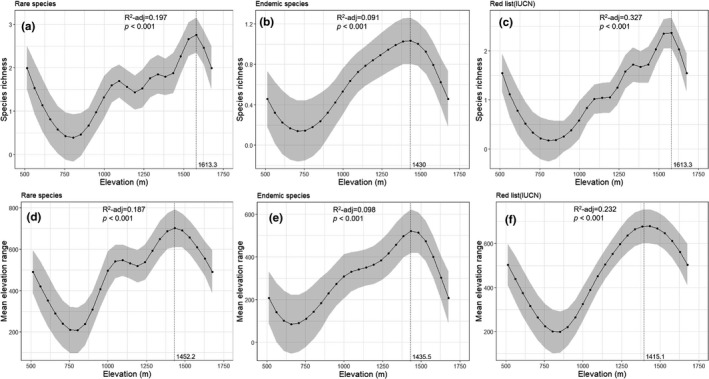
Elevation patterns in species richness of (a) rare species, (b) endemic species, and (c) red list (IUCN), and mean elevation range of (d) rare species, (e) endemic species, and (f) red list (IUCN) species on the Seorak Mountain

Rare, endemic, and red list species with phytogeographic affinities intermittently appeared on the Seorak Mountain from 500 m; in contrast to the elevation patterns of the life forms or total species, the rare species increased continuously from 1,613.3 m (Figure 5a), and endemic species exhibited a positive unimodal pattern at 1,430 m (Figure 5b). Red list species showed elevation patterns similar to that of rare species (Figure 5c). Furthermore, rare and endemic species were almost absent below 800 m on the Seorak Mountain (Figure 5). The mean elevation range was slightly adjusted to the range size compared to species richness and it exhibited a pattern similar to that of the species richness elevation.

## 4 DISCUSSION

The results of the empirical data were similar to that in previous studies for various taxa (e.g., birds, land snails, and fish); they were unimodal or multimodal in richness along the elevation gradients (Carvajal‐Quintero et al., 2015; Liew et al., 2010; Pan et al., 2016). The mid‐peak in richness was followed by a hump at elevations between 900 and 1,100 m (Figure 2). The relationship between elevation and species richness in Seorak Mountain could be understood based on the unimodal pattern attributed to the accumulation of species with a wide range of sizes and the emergence of various species (Figure 3).

In tropical and subtropical mountains, unimodal patterns of organisms are common and are more likely to appear, because of peak diversity below the mid‐point of elevation (Cirimwami et al., 2019; Feng et al., 2016; Guo et al., 2013; Rahbek, 2005; Zhou et al., 2019). Mountains always exhibit a larger altitude range and longer climatic gradients; and therefore, they generally have unimodal patterns.

The significant unimodal pattern in the proportion of woody plants in relatively low mountain areas, including Seorak Mountain, reflects their physiological adaptations. The pattern of these woody plants suggests that a strong boundary effect can result in a unimodal pattern (Colwell et al., 2005; Feng et al., 2016; Vetaas & Grytnes, 2002). Seorak Mountain is a low mountain with an altitude of 1,708 m; however, its treeline begins at 1,500 m, above which characteristic dwarf and arctic‐alpine plants appear (Kim, Lim, et al., 2019; Kong, 2002). This boundary effect can also be seen in the mean elevation range size. The mean elevation range size mostly peaked below 1,500 m and showed a single‐mode pattern (Figure 4). Therefore, the boundary effect could induce a mean range size unimodal trend in mid‐elevation areas, and a similar unimodal trend in life forms with a wide elevation range.

Furthermore, the presence or absence of rare and endemic species (maximum peaks at 1,613.3 and 1,430 m, respectively; Figure 5) greatly influences the altitude patterns of the total species richness (elevation 900 to 1,100 m; Figure 2a,b). When rare and endemic species were excluded, the species richness followed the unimodal rule from approximately 1,000 m and with higher altitudes. This is possibly because of the shorter growing seasons, lower temperatures, lower mass circulation, and treeline‐like environments (i.e., physical environments such as hard rock formations and physiological constraints due to extreme climatic conditions). The region around the Daecheongbong Peak of Seorak Mountain has strong winds and a rocky terrain; this will benefit species with a small distribution range such as rare or endemic species (Kim et al., 2017; Kim, Lim, et al., 2019).

In the Himalayas (Bhattarai & Vetaas, 2006; Feng et al., 2016; Vetaas & Grytnes, 2002), Andes (Cuesta et al., 2017; Hutter et al., 2013), and various African mountain ranges (Cirimwami et al., 2019; Zhou et al., 2019), the species richness of rare and endemic species continuously increased at higher altitudes, a pattern similar to that in most mountains. In the Himalayan Mountains of Nepal, the number of endemic species increases with elevation gradients up to an altitude of 4,200 m (Vetaas & Grytnes, 2002), and rare and endemic species in Korea's Seorak (maximum peak 1,708 m) increased at 1,613.3 and 1,430 m, respectively (Figure 5). The physiological adaptations of the plants could have reduced the range size of endemic species or rare species at a range different from that for the general species, particularly in the lowlands. Therefore, endemic and rare species could peak at altitudes that are higher than that for the total species.

Tropical or endemic species exhibited a small elevation range size (Zhou et al., 2019). Similarly, on Seorak Mountain (Figure 5), most rare or endemic species remaining after the Pleistocene Epoch showed a small elevation range size (Chung et al., 2017; Kong, 2002, 2004). At higher altitudes, the range size of species in the assembly is explained as the result of individuals having to withstand extreme climatic conditions at higher altitudes (Feng et al., 2016; Gaston, 1996; Gaston & Chown, 1999; Morin & Lechowicz, 2011). Therefore, even if the overall elevation range is small, the species pool has a similar shape (ecosystem), and the adjusted range size can be predicted for each species.

Both the native plant species in tropical regions (Zhou et al., 2019) and the species with geographical isolation and distribution to a specific region have phytogeographical affinities. The phytogeographic results are included for mountain regions of specific heights and distributions; realistic predictions of ERR must be assessed in the various mountain regions to improve its relevance.

A strong support for the range–elevation relationship predicted by the ERR was observed in herbs, rare, endemic, and red list species (Figures 4e and 5d–f). The boundary effects due to environmental or climatic conditions could cause a trend of decreasing mean elevation range at high elevation regions (Bhattarai & Vetaas, 2006; Feng et al., 2016; Vetaas & Grytnes, 2002). This study shows support for the ERR with regard to the increasing trend in the elevation relationship of the range size of herbs, rare, endemic, and red list species; however, the boundary effect was not strongly observed. The proportion of endemic and rare species increasing along the elevation gradient can affect the relationship between the mean elevation range and elevation of species assemblages (Pottier et al., 2013; Vetaas & Grytnes, 2002). On Seorak Mountain, rare and endemic species were distributed continuously and they appeared at high elevation gradients (species included *Adenophora grandiflora*, *Weigela subsessilis*, *Lonicera subsessilis*, *Viola diamantiaca*, *Syringa wolfii*, *Rodgersia podophylla*, *Smilacina bicolor*, *Patrinia saniculifolia*, and *P. pumila*, which were from different life‐form groups).

Compared to the narrowly distributed species, widely distributed species always have a wider range and adaptability. For example, a widely distributed species always has a wide range and strong tolerance compared to a narrowly distributed species (Donohue et al., 2010; Gaston & Spicer, 2001; Santamaría, 2002). However, it does not necessarily mean that species with a wide distribution can adapt even at the peak of elevation. Relatively, species with a narrow distribution range physiologically and ecologically adapt to an extreme climate or environments over a long period and then emerge or be observed at specific elevation ranges. This is because, considering the boundary effect, support for ERR could depend on phytogeographic affinities.

## 5 CONCLUSIONS

The altitude range of the herbs, rare, endemic, and red list species was significantly higher than that of woody plants. Particularly, the rare, endemic, and red list species can withstand extreme climatic conditions through physiological adaptations, as their ranges reached the highest elevation, and therefore, ERR can be applied to them. However, the hard boundary effect in this region consistently supported different life forms (i.e., trees, shrubs, lianas, pteridophytes, and woody species). Overall, the ERR was inconsistent between plants of different life forms in the same region. In conclusion, even though the elevation range of Seorak mountain is small compared to the world's highest mountain, the species pool has a similar shape. That is, possible to predict the adjusted range‐size for each species.

## CONFLICT OF INTEREST

The authors declare that they have no conflict of interest.

## AUTHOR CONTRIBUTIONS

**Mi‐Hyun Lee:** Data curation (equal); Formal analysis (equal); Software (equal); Visualization (lead); Writing‐original draft (lead); Writing‐review & editing (lead). **Ju Hyeon Song:** Investigation (equal); Writing‐review & editing (equal). **Byeon Seong Yeob:** Formal analysis (equal); Investigation (equal). **Jeong Eun Lee:** Data curation (equal); Investigation (equal). **Ho Jin Kim:** Investigation (equal); Writing‐review & editing (equal). **Seung‐Beom Chae:** Investigation (equal); Visualization (equal). **Chung Weon Yun:** Funding acquisition (lead); Project administration (equal); Resources (equal); Supervision (lead). **Ji‐Dong Kim:** Conceptualization (lead); Formal analysis (lead); Investigation (equal); Methodology (lead); Project administration (lead); Software (lead); Supervision (equal); Writing‐original draft (equal); Writing‐review & editing (equal).

## Data Availability

All data for analysis are available at Dryad (https://doi.org/10.5061/dryad.d51c5b02x).

## References

[ece38033-bib-0001] Addo‐Bediako, A., Chown, S. L., & Gaston, K. J. (2000). Thermal tolerance, climatic variability and latitude. Proceedings of the Royal Society of London. Series B: Biological Sciences, 267, 739–745. 10.1098/rspb.2000.1065 10819141 PMC1690610

[ece38033-bib-0002] Almeida‐Neto, M., Machado, G., Pinto‐da‐Rocha, R., & Giaretta, A. A. (2006). Harvestman (Arachnida: Opiliones) species distribution along three Neotropical elevational gradients: An alternative rescue effect to explain Rapoport's rule? Journal of Biogeography, 33, 361–375. 10.1111/j.1365-2699.2005.01389.x

[ece38033-bib-0003] Bergamin, R. S., Seger, G. D. S., Carlucci, M. B., Molz, M., Mello, R. S. P., Martins, R., Jarenkow, J. A., Brack, P., Müller, S. C., & Duarte, L. D. S. (2021). Elevational shifts in phylogenetic diversity of angiosperm trees across the subtropical Brazilian Atlantic Forest. Austral Ecology, 46(3), 486–495. 10.1111/aec.12996

[ece38033-bib-0004] Bhattarai, K. R., & Vetaas, O. R. (2006). Can Rapoport's rule explain tree species richness along the Himalayan elevation gradient, Nepal? Diversity and Distributions, 12, 373–378. 10.1111/j.1366-9516.2006.00244.x

[ece38033-bib-0005] Braun‐Blanquet, J. (2013). Pflanzensoziologie Grundzüge der Vegetationskunde. Springer.

[ece38033-bib-0006] Brown, J. H., Stevens, G. C., & Kaufman, D. M. (1996). The geographic range: Size, shape, boundaries, and internal structure. Annual Review of Ecology and Systematics, 27, 597–623. 10.1146/annurev.ecolsys.27.1.597

[ece38033-bib-0007] Carvajal‐Quintero, J. D., Escobar, F., Alvarado, F., Villa‐Navarro, F. A., Jaramillo‐Villa, Ú., & Maldonado‐Ocampo, J. A. (2015). Variation in freshwater fish assemblages along a regional elevation gradient in the northern Andes, Colombia. Ecology and Evolution, 5, 2608–2620. 10.1002/ece3.1539 26257874 PMC4523357

[ece38033-bib-0008] Chan, W. P., Chen, I. C., Colwell, R. K., Liu, W. C., Huang, C. Y., & Shen, S. F. (2016). Seasonal and daily climate variation have opposite effects on species elevational range size. Science, 351, 1437–1439. 10.1126/science.aab4119 27013729

[ece38033-bib-0009] Chung, M. Y., López‐Pujol, J., & Chung, M. G. (2017). The role of the Baekdudaegan (Korean Peninsula) as a major glacial refugium for plant species: A priority for conservation. Biological Conservation, 206, 236–248. 10.1016/j.biocon.2016.11.040

[ece38033-bib-0010] Cirimwami, L., Doumenge, C., Kahindo, J. M., & Amani, C. (2019). The effect of elevation on species richness in tropical forests depends on the considered lifeform: Results from an East African mountain forest. Tropical Ecology, 60, 473–484. 10.1007/s42965-019-00050-z

[ece38033-bib-0011] Colwell, R. K., & Hurtt, G. C. (1994). Nonbiological gradients in species richness and a spurious Rapoport effect. The American Naturalist, 144, 570–595. 10.1086/285695

[ece38033-bib-0012] Colwell, R. K., Rahbek, C., & Gotelli, N. J. (2005). The mid‐domain effect: There's a baby in the bathwater. The American Naturalist, 166, E149–E154. 10.1086/491689

[ece38033-bib-0013] Cuesta, F., Muriel, P., Llambí, L. D., Halloy, S., Aguirre, N., Beck, S., Carilla, J., Meneses, R. I., Cuello, S., Grau, A., Gámez, L. E., Irazábal, J., Jácome, J., Jaramillo, R., Ramírez, L., Samaniego, N., Suárez‐Duque, D., Thompson, N., Tupayachi, A., … Gosling, W. D. (2017). Latitudinal and altitudinal patterns of plant community diversity on mountain summits across the tropical Andes. Ecography, 40, 1381–1394. 10.1111/ecog.02567

[ece38033-bib-0014] Donohue, K., Rubio de Casas, R. C., Burghardt, L., Kovach, K., & Willis, C. G. (2010). Germination, post germination adaptation, and species ecological ranges. Annual Review of Ecology, Evolution, and Systematics, 41, 293–319. 10.1146/annurev-ecolsys-102209-144715

[ece38033-bib-0015] Elsen, P. R., & Tingley, M. W. (2015). Global mountain topography and the fate of montane species under climate change. Nature Climate Change, 5, 772–776. 10.1038/nclimate2656

[ece38033-bib-0016] Feeley, K. J., Rehm, E. M., & Machovina, B. (2012). Perspective: The responses of tropical forest species to global climate change: Acclimate, adapt, migrate, or go extinct? Frontiers of Biogeography, 4, 69–84. 10.21425/F5FBG12621

[ece38033-bib-0017] Feng, J., Hu, X., Wang, J., & Wang, Y. (2016). Support for the elevational Rapoport's rule among seed plants in Nepal depends on biogeographical affinities and boundary effects. Ecology and Evolution, 6, 7246–7252. 10.1002/ece3.2473 28725394 PMC5513255

[ece38033-bib-0018] Fleishman, E., Austin, G. T., & Weiss, A. D. (1998). An empirical test of Rapoport's rule: Elevational gradients in montane butterfly communities. Ecology, 79, 2482–2493. 10.1890/0012-9658(1998)079#;2482:AETORS#;2.0.CO;2

[ece38033-bib-0019] Freeman, B. G., & Class Freeman, A. M. (2014). Rapid upslope shifts in New Guinean birds illustrate strong distributional responses of tropical montane species to global warming. Proceedings of the National Academy of Sciences of the United States of America, 111, 4490–4494. 10.1073/pnas.1318190111 24550460 PMC3970498

[ece38033-bib-0020] Gaston, K. J. (1996). Species‐range‐size distributions: Patterns, mechanisms and implications. Trends in Ecology and Evolution, 11, 197–201. 10.1016/0169-5347(96)10027-6 21237808

[ece38033-bib-0021] Gaston, K. J. (1998). Species‐range size distributions: Products of speciation, extinction and transformation. Philosophical Transactions of the Royal Society of London. Series B: Biological Sciences, 353, 219–230. 10.1098/rstb.1998.0204

[ece38033-bib-0022] Gaston, K. J., & Chown, S. L. (1999). Elevation and climatic tolerance: A test using dung beetles. Oikos, 86, 584–590. 10.2307/3546663

[ece38033-bib-0023] Gaston, K. J., & Spicer, J. I. (2001). The relationship between range size and niche breadth: A test using five species of *Gammarus* (Amphipoda). Global Ecology and Biogeography, 10, 179–188. 10.1046/j.1466-822x.2001.00225.x

[ece38033-bib-0024] Guerrero, P. C., Durán, A. P., & Walter, H. E. (2011). Latitudinal and altitudinal patterns of the endemic cacti from the Atacama desert to Mediterranean Chile. Journal of Arid Environments, 75, 991–997. 10.1016/j.jaridenv.2011.04.036

[ece38033-bib-0025] Guo, Q., Kelt, D. A., Sun, Z., Liu, H., Hu, L., Ren, H., & Wen, J. (2013). Global variation in elevational diversity patterns. Scientific Reports, 3, 3007. 10.1038/srep03007 24157658 PMC6505670

[ece38033-bib-0026] Harte, J., & Shaw, R. (1995). Shifting dominance within a montane vegetation community: Results of a climate‐warming experiment. Science, 267, 876–880. 10.1126/science.267.5199.876 17813919

[ece38033-bib-0027] Hastie, T. J., & Tibshirani, R. J. (1990). Generalized additive models. Chapman & Hall. 10.1177/096228029500400302 8548102

[ece38033-bib-0028] Hernández‐Rojas, A. C., Kluge, J., Krömer, T., Carvajal‐Hernández, C., Silva‐Mijangos, L., Miehe, G., Lehnert, M., Weigand, A., & Kessler, M. (2020). Latitudinal patterns of species richness and range size of ferns along elevational gradients at the transition from tropics to subtropics. Journal of Biogeography, 47, 1383–1397. 10.1111/jbi.13841

[ece38033-bib-0029] Hutter, C. R., Guayasamin, J. M., & Wiens, J. J. (2013). Explaining Andean megadiversity: The evolutionary and ecological causes of glassfrog elevational richness patterns. Ecology Letters, 16, 1135–1144. 10.1111/ele.12148 23802805

[ece38033-bib-0030] IUCN . (2020). The IUCN Red List of Threatened Species. Version 2020‐3. https://www.iucnredlist.org

[ece38033-bib-0031] Kessler, M. (2000). Elevational gradients in species richness and endemism of selected plant groups in the central Bolivian Andes. Plant Ecology, 149, 181–193. 10.1023/A:1026500710274

[ece38033-bib-0032] Kim, J. D., Lim, J. H., & Yun, C. W. (2019). Dynamics of *Abies nephrolepis* seedlings in relation to environmental factors in Seorak Mountain, South Korea. Forests, 10, 702. 10.3390/f10080702

[ece38033-bib-0033] Kim, J. D., Park, G. E., Lim, J. H., & Yun, C. W. (2017). Phytosociological community type classification and flora of vascular plants for the forest vegetation of Daecheongbong area in Mt. Seorak. Journal of Korean Forest Science, 106, 130–149. 10.14578/jkfs.2017.106.2.130

[ece38033-bib-0034] Kim, J. Y., Seo, C., Hong, S., Lee, S., & Eo, S. H. (2019). Altitudinal range‐size distribution of breeding birds and environmental factors for the determination of species richness: An empirical test of altitudinal Rapoport's rule and non‐directional rescue effect on a local scale. PLoS One, 14, e0203511. 10.1371/journal.pone.0203511 30682009 PMC6347176

[ece38033-bib-0035] Kong, W. S. (2002). Species composition and distribution of Korean Alpine plants. Journal of the Korean Geographical Society, 37, 357–370.

[ece38033-bib-0036] Kong, W. S. (2004). Species composition and distribution of native Korean conifers. Journal of the Korean Geographical Society, 39, 528–543.

[ece38033-bib-0037] Korea Forest Service . (2010a). Korea Biodiversity Information System. http://www.nature.go.kr/

[ece38033-bib-0038] Korea Forest Service . (2010b). Korea Plant Names Index Committee . http://www.nature.go.kr/kpni/

[ece38033-bib-0039] Korea National Arboretum . (2008). Rare plants data book in Korea. Geobook.

[ece38033-bib-0040] Korea National Arboretum . (2010). 300 Target plants adaptable to climate change in the Korea peninsula. Theulmunhwa.

[ece38033-bib-0041] Kreft, H., Jetz, W., Mutke, J., & Barthlott, W. (2010). Contrasting environmental and regional effects on global pteridophyte and seed plant diversity. Ecography, 33, 408–419. 10.1111/j.1600-0587.2010.06434.x

[ece38033-bib-0042] Külköylüoğlu, O., Sari, N., Akdemir, D., Yavuzatmaca, M., & Altinbağ, C. (2012). Distribution of sexual and asexual Ostracoda (Crustacea) from different altitudinal ranges in the Ordu region of Turkey: Testing the rapoport rule. High Altitude Medicine and Biology, 13, 126–137. 10.1089/ham.2011.1111 22724616

[ece38033-bib-0043] Kwon, T. S., Kim, S. S., & Chun, J. H. (2014). Pattern of ant diversity in Korea: An empirical test of Rapoport's altitudinal rule. Journal of Asia‐Pacific Entomology, 17, 161–167. 10.1016/j.aspen.2013.12.006

[ece38033-bib-0044] Lee, T. B. (2003). Coloured flora of Korea. Hyangmunsa.

[ece38033-bib-0045] Liew, T. S., Schilthuizen, M., & Lakim, M. (2010). The determinants of land snail diversity along a tropical elevational gradient: Insularity, geometry and niches. Journal of Biogeography, 37, 1071–1078. 10.1111/j.1365-2699.2009.02243.x

[ece38033-bib-0046] Lomolino, M. V., Riddle, B. R., Brown, J. H., & Brown, J. H. (2006). Biogeography (No. QH84 L65 2006). Sinauer Associates.

[ece38033-bib-0048] McCain, C. M., & Bracy Knight, K. (2013). Elevational Rapoport's rule is not pervasive on mountains. Global Ecology and Biogeography, 22, 750–759. 10.1111/geb.12014

[ece38033-bib-0049] McCain, C. M., & Colwell, R. K. (2011). Assessing the threat to montane biodiversity from discordant shifts in temperature and precipitation in a changing climate. Ecology Letters, 14, 1236–1245. 10.1111/j.1461-0248.2011.01695.x 21981631

[ece38033-bib-0050] Morin, X., & Lechowicz, M. J. (2011). Geographical and ecological patterns of range size in North American trees. Ecography, 34, 738–750. 10.1111/j.1600-0587.2010.06854.x

[ece38033-bib-0051] Ogwu, M. C., Takahashi, K., Dong, K., Song, H. K., Moroenyane, I., Waldman, B., & Adams, J. M. (2019). Fungal elevational Rapoport pattern from a High Mountain in Japan. Scientific Reports, 9, 6570. 10.1038/s41598-019-43025-9 31024040 PMC6484014

[ece38033-bib-0052] Oommen, M. A., & Shanker, K. (2005). Elevational species diversity patterns emerge from multiple local mechanisms in Himalayan woody plants. Ecology, 86, 3039–3047. 10.1890/04-1837

[ece38033-bib-0053] Pan, X., Ding, Z., Hu, Y., Liang, J., Wu, Y., Si, X., Guo, M., Hu, H., & Jin, K. (2016). Elevational pattern of bird species richness and its causes along a central Himalaya gradient, China. PeerJ, 4, e2636. 10.7717/peerj.2636 27833806 PMC5101612

[ece38033-bib-0054] Pintor, A. F., Schwarzkopf, L., & Krockenberger, A. K. (2015). Rapoport's rule: Do climatic variability gradients shape range extent? Ecological Monographs, 85, 643–659. 10.1890/14-1510.1

[ece38033-bib-0055] Pottier, J., Dubuis, A., Pellissier, L., Maiorano, L., Rossier, L., Randin, C. F., Vittoz, P., & Guisan, A. (2013). The accuracy of plant assemblage prediction from species distribution models varies along environmental gradients. Global Ecology and Biogeography, 22, 52–63. 10.1111/j.1466-8238.2012.00790.x

[ece38033-bib-0056] R Core Team . (2020). R: A language and environment for statistical computing. R Foundation for Statistical Computing. https://www.R‐project.org/

[ece38033-bib-0057] Rahbek, C. (1995). The elevational gradient of species richness: A uniform pattern? Ecography, 18, 200–205. 10.1111/j.1600-0587.1995.tb00341.x

[ece38033-bib-0058] Rahbek, C. (1997). The relationship among area, elevation, and regional species richness in neotropical birds. American Naturalist, 149, 875–902. 10.1086/286028 18811253

[ece38033-bib-0059] Rahbek, C. (2005). The role of spatial scale and the perception of large‐scale species‐richness patterns. Ecology Letters, 8, 224–239. 10.1111/j.1461-0248.2004.00701.x

[ece38033-bib-0060] Raunkiær, C. (1934). The life forms of plants and statistical plant geography. Oxford University Press.

[ece38033-bib-0061] Ribas, C. R., & Schoereder, J. H. (2006). Is the Rapoport effect widespread? Null models revisited. Global Ecology and Biogeography, 15, 614–624. 10.1111/j.1466-8238.2006.00265.x

[ece38033-bib-0062] Rohner, P. T., Bächli, G., Pollini Paltrinieri, L., Duelli, P., Obrist, M. K., Jochmann, R., & Blanckenhorn, W. U. (2015). Distribution, diversity gradients and Rapoport's elevational rule in the black scavenger flies of the Swiss Alps (Diptera: Sepsidae). Insect Conservation and Diversity, 8, 367–376. 10.1111/icad.12114

[ece38033-bib-0063] Sanders, N. J. (2002). Elevational gradients in ant species richness: Area, geometry, and Rapoport's rule. Ecography, 25, 25–32. 10.1034/j.1600-0587.2002.250104.x

[ece38033-bib-0064] Santamaría, L. (2002). Why are most aquatic plants widely distributed? Dispersal, clonal growth and small‐scale heterogeneity in a stressful environment. Acta Oecologica, 23, 137–154. 10.1016/S1146-609X(02)01146-3

[ece38033-bib-0065] Smith, A. R. (1993). Phytogeographic principles and their use in understanding fern relationships. Journal of Biogeography, 20, 255–264. 10.2307/2845633

[ece38033-bib-0066] Stevens, G. C. (1992). The elevational gradient in altitudinal range: An extension of Rapoport's latitudinal rule to altitude. American Naturalist, 140, 893–911. 10.1086/285447 19426029

[ece38033-bib-0067] Tomašových, A., Kennedy, J. D., Betzner, T. J., Kuehnle, N. B., Edie, S., Kim, S., Supriya, K., White, A. E., Rahbek, C., Huang, S., Price, T. D., & Jablonski, D. (2016). Unifying latitudinal gradients in range size and richness across marine and terrestrial systems. Proceedings of the Royal Society of London. Series B: Biological Sciences, 283, 20153027. 10.1098/rspb.2015.3027 27147094 PMC4874701

[ece38033-bib-0068] Trigas, P., Panitsa, M., & Tsiftsis, S. (2013). Elevational gradient of vascular plant species richness and endemism in Crete–the effect of post‐isolation mountain uplift on a continental island system. PLoS One, 8, e59425. 10.1371/journal.pone.0059425 23555031 PMC3595250

[ece38033-bib-0069] Vetaas, O. R., & Grytnes, J. A. (2002). Distribution of vascular plant species richness and endemic richness along the Himalayan elevation gradient in Nepal. Global Ecology and Biogeography, 11, 291–301. 10.1046/j.1466-822X.2002.00297.x

[ece38033-bib-0070] Wang, Z., Tang, Z., & Fang, J. (2007). Altitudinal patterns of seed plant richness in the Gaoligong Mountains, southeast Tibet, China. Diversity and Distributions, 13, 845–854. 10.1111/j.1472-4642.2007.00335.x

[ece38033-bib-0071] Wu, Y., Colwell, R. K., Han, N., Zhang, R., Wang, W., Quan, Q., Zhang, C., Song, G., Qu, Y., & Lei, F. (2014). Understanding historical and current patterns of species richness of babblers along a 5000‐m subtropical elevational gradient. Global Ecology and Biogeography, 23, 1167–1176. 10.1111/geb.12197

[ece38033-bib-0072] Zhou, Y., Ochola, A. C., Njogu, A. W., Boru, B. H., Mwachala, G., Hu, G., Xin, H., & Wang, Q. (2019). The species richness pattern of vascular plants along a tropical elevational gradient and the test of elevational Rapoport's rule depend on different life‐forms and phytogeographic affinities. Ecology and Evolution, 9, 4495–4503. 10.1002/ece3.5027 31031922 PMC6476750

